# Review of Session 6: Medical Physics

**DOI:** 10.1093/jrr/rrt225

**Published:** 2014-03

**Authors:** Shigekazu Fukuda

**Affiliations:** National Institute of Radiological Sciences, Chiba, Japan

**Keywords:** heavy ion, fragmentation, neutron, lineal energy

## Abstract

Medical physics is very important in carbon ion radiotherapy, as it is in conventional radiotherapy using X-rays and in estimation of exposed dose in the space environment. High-energy ion beams such as carbon beams have physical characteristics such as the Bragg curve, high LET, and nuclear reactions producing fragmentations. Therefore, understanding these properties well is essential for further development of carbon radiotherapy and manned space activity. We invited, therefore, the following six presentations relevant to issues ranging from the measurement of fragmentations, lineal energy distributions using the microdosimetric approach, and neutron dose with active beam delivery of carbon-ion therapy, to the depth–dose distribution of various ions inside a human head phantom.

- ‘Experimental investigation of secondary ions for carbon ion therapy' was presented by Dr Martisikova. She and her colleagues have been trying to develop a system with Timepix (a pixelized semiconductor detector) that can monitor dose distribution in a patient online. This system makes use of secondary ions produced in prompt nuclear processes in the patient and registers the secondary ions emerging from the irradiated target to Timepix. This method is expected to be less affected by washout than when measuring tissue activation with a PET camera. This group performed the experiments with a homogeneous phantom and an anthropomorphous phantom at the Heidelberg Ion Beam Therapy Facility and analyzed the dependencies of the measured distributions of secondary ions on the beam setting. They reported that the application of beam energies with range differences in water of 1 mm could be distinguished in the homogeneous phantom. These results sound promising for further development of practical therapy monitoring systems.
- ‘Measurement of neutron ambient dose equivalent in carbon-ion radiotherapy with active scanned beam' was presented by Dr Yonai. It is essential to be able to estimate the secondary cancer risk, especially for a young patient, even though the out-of-field dose due to secondary neutrons and leakage of charged particles is considerably lower than the in-field dose in ion beam radiotherapy. Dr Yonai and colleagues measured the neutron ambient dose equivalents in carbon-ion radiotherapy with active beams using a rem meter at the new treatment research facility in NIRS and compared these results with those for passive beams. They showed that the neutron dose with active beams was a maximum of ∼ 14% of that with passive beams for equivalent target volumes. Further investigation is needed into the difference in clinical outcomes, including secondary cancer risk, between carbon-ion radiotherapy with active beams and that with passive beams.
- ‘Ground based radiation field simulation of the MATROSHKA experiment: Physical and Biological Experiments for Radiation Risk Assessment – PARTII' was presented by Dr Berger. In order to support and extend space radiation research, the main objective of the experiment of Dr Berger and his colleagues was a detailed study of the depth–dose radiation distribution inside a human head phantom for selected heavy ions and energies. As a controlled ground-based simulation of the space experiment, they irradiated the phantom at GSI with nickel and iron ions with a set-up that allowed for intercomparison of the data received by a number of detector systems measuring directly and simultaneously. They reported that the relative growth of HEK-ptdTomato-N1 cells 100 h after exposure to nickel and iron ions in one slice of the phantom head showed that survival of cells is highest in the back of the head and lowest in the forehead region, with the beam entering from the nose.
- ‘Study of carbon ion fragmentation in different materials using a highly flexible set-up' was presented by Dr Martisikova. She and her colleagues applied Timepix (described in the second presentation) to an investigation of the fragmentation of carbon nuclei in tissue, which is an important issue in carbon-ion beam therapy treatment planning. They performed the experiment at Heidelberg Ion Beam Therapy Facility and studied fragmentation in a range of materials. They claimed the system could distinguish primary carbon ions and secondary H, He, Li, Be and B ions (and also electrons) by using pattern recognition analysis of the signal parameters. Their study demonstrated the potential of their system, together with Timepix, for distinguishing fragments in mixed particle fields for detailed analysis of ^12^C-fragmentation in different targets. We expect that their novel method will contribute to determining the beam quality of carbon-ion therapeutic beams, which till now has not been rigorously defined.
- ‘Systematic measurements of lineal energy distributions of various energetic heavy ion beams using wall-less tissue equivalent proportional counter' was presented by Dr Tsuda. Dr Tsuda and colleagues have systematically measured the distributions of lineal energy, *yf(y)*, for various energetic heavy ions of H, ^2^He, ^12^C, ^28^Si and ^56^Fe using a wall-less tissue equivalent, and compared their results with those obtained from simulation codes such as PHITS. They report that there were differences between the shape of the measured *yf(y)* distributions and the calculated data from the microdosimetric function of the PHITS code. Because information about the deposited energy distribution in a microscopic site is essential for understanding the relative biological effectiveness (RBE) and determining the radiation quality of therapeutic carbon-ions beams, we hope for further development and progression in both measurement and simulations.
- ‘Fragmentation of ^56^Fe on C, Al and CH_2_ targets at 471 A MeV' was presented by Dr Zhang. Dr Zhang and colleagues measured the total charge-change cross sections, the partial cross sections of fragmentation productions, the emission angles and the transverse momentum distribution of the fragmentation of ^56^Fe on Al, C, CH_2_ and H targets at 471 A MeV. These basic physical quantities are very important for understanding heavy ion therapy and exposure dose in space, as well as nuclear physics. They also reported that the improved quantum molecular model (ImQMD) combined with the GEMNI model can well represent the production of charged projectile fragments and the emission angles and transverse momentum distributions of fragments. Their results agreed well with those obtained from the ImQMD alone.

These six presentations included brand new results and contributions to recent development and progression in medical physics research. We hope that increased exchange and sharing of information and basic data (such as microdosimetric quantities) among the research groups relevant to heavy-ion therapy, the space environment, and radiation detectors will encourage further activity in medical physics research.

## FUNDING

